# Sex-Based Differences in the Quality of Life of Elderly Koreans With Chronic Musculoskeletal Pain

**DOI:** 10.3390/ijerph17030743

**Published:** 2020-01-23

**Authors:** Hyesun Jeong, Yoonju Lee

**Affiliations:** 1 Department of Nursing, Graduate School, Pusan National University, Yangsan 50612, Korea; pointsun@naver.com; 2 College of Nursing, Pusan National University, Yangsan 50612, Korea

**Keywords:** chronic musculoskeletal pain, aged, sex, quality of life

## Abstract

In this study, we constructed a structural equation model (SEM) for predicting the quality of life (QOL) in elderly Koreans with chronic musculoskeletal pain (CMP) and examined the differences between sexes. Data were earlier collected in a prior study of 307 participants (101 men and 206 women) with CMP, aged 65 years and above, who used geriatric welfare centers located in two cities. The effects of pain, functional limitation, perceived health status, pain coping, and social support on the QOL were estimated with a multigroup SEM. For both men and women, the results show sequential causality from pain to functional limitation, perceived health status, and QOL. However, the relationships among pain, pain coping, functional limitation, and QOL differ between men and women. The multigroup SEM provides a better understanding of the sex differences in the QOL of elderly with CMP. The results suggest that in order to improve QOL among the elderly with CMP, a customized strategy should be applied that takes into account differences between the sexes.

## 1. Introduction

The aging of the population is one of the most important problems globally. Aging leads to an increased incidence of chronic disease, especially musculoskeletal disease (MSD). As the prevalence of MSD increases with age, 20% to 30% of people across the globe are living with painful musculoskeletal impairments [1]. The prevalence of MSD in the Korean elderly aged 65 years or over increases yearly, and has been estimated to reach 70.2% by 2017, with a of 2.3-fold increase expected by 2040 [2,3]. MSD often occurs in the joints and most commonly in the knee, shoulder, lumbar, and cervical areas [4]. One major MSD symptom in the elderly is chronic pain, which may be persistent or recurrent, due to the disease directly affecting bones, joints, muscles, or related soft tissues [5]. Chronic musculoskeletal pain (CMP) is a major health problem for the elderly, experienced by 83% of the elderly in the community [6].

The prevalence, symptoms, and responses to symptoms differ between the sexes. According to the World Health Organization (WHO) nationwide study on aging (SAGE), a comparison of MSD prevalence between men and women showed that general chronic back pain and joint pain was more prevalent among women than men [7]. Women exhibit a lower threshold for musculoskeletal pain than men, making them more sensitive to pain [8,9]. Pain-related worries were more common in women than men, with subjective pain and discomfort being higher in women [10]. In addition, CMP-related depression was significantly higher in women than men [11]. In a large longitudinal study of the elderly community in Singapore, differences between the sexes in daily performance and perceived health status variables were observed [12].

Pain coping strategies are the specific set of behaviors that individuals use to control and emotionally respond to their pain [13]. How a person copes with pain regulates the physical and mental factors that ultimately affect their quality of life (QOL) [14]. However, a systematic literature review of the analysis of sex differences in coping styles for chronic pain showed that women are passive and maladaptive, whereas men are more adaptive [15]. The absence of a social support can be a leading cause of the deterioration of psychological as well as physical health [16,17] while strong social support exhibits a positive effect on the health-related QOL of the elderly [18]. Compared to men, women tend to underestimate their ability to cope, and obtain social support from their surroundings [12].

Older people with CMP exhibit lower QOLs compared to those without [9], and those with MSD report significantly lower QOLs compared to those with cardiovascular or endocrine chronic diseases [19]. Since the health-related QOL of elderly people who experience CMP is an important issue for health promotion in the elderly, identifying the various factors influencing improvements in QOL is necessary. 

Wilson and Cleary proposed a conceptual model for health-related QOL, including the clinical and social science paradigm [20]. Based on Wilson and Cleary’s model, some previous studies have attempted to explain the QOL of elderly people with CMP using some dimensions or variables, such as pain, capability to perform the tasks of daily living, and physical activity limitation [21], as well as psychological aspects, such as depression, anxiety, and powerlessness [22]. In a prior study, the authors examined the aspects that affect the QOL of elderly with CMP, including demographic characteristics, pain characteristics, functional limitations and perceived health status, pain coping, and social support [23]. The results showed that high levels of education, low pain, low functional limitations, use of accommodative coping with pain, a high perceived health status, and strong family and friend support are factors related to improvements in QOL. However, the study used multiple regression analyses, which cannot evaluate the direct and indirect effects of the variable associated with the QOL and, therefore, cannot identify the structural relationships between predictors. In other words, the authors found that pain, functional limitations, pain management, perceived health status, and social support all affect QOL. However, the regression coefficients of each variable only indicate the influence when the other variables are adjusted, so the relationship between the variables and the effect of the relationship cannot be identified. Only the explanatory power explained by the total independent variables included in the multiple regression model can be determined; the specific relationships between the variables and the direct and indirect effects on the dependent variables cannot be calculated. 

The structural equation model (SEM) overcomes these limitations of multiple regression. One principal difference with SEM is that a construct that acts as an independent variable in one relationship can be the dependent variable in another relationship [24]. For instance, the perceived health status in multiple regression analysis is an independent variable for QOL; in the hypothetical model in this study, it is a dependent variable of pain coping and social support, and also a mediating variable in the relationship between pain coping and social support and QOL. Hence, both the direct and indirect effects of perceived health status on QOL could be examined.

A review of the literature did not reveal the structure of the variables affecting QOL of the elderly who experience CMP, and it was not clear whether there were any differences between sexes. The purpose of this study was to empirically test the theory-based hypothetical model and to identify sex differences to explain the relationship between variables related to the QOL of elderly people who experience CMP using multigroup structural equation model analysis.

## 2. Materials and Methodology

### 2.1. Hypothetical Model

**Figure 1:**
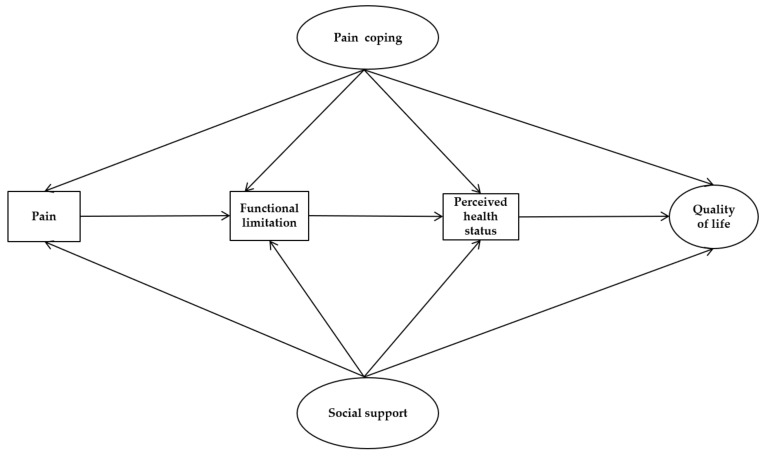
Hypothetical model of the study.

We constructed a hypothetical model based on Wilson and Cleary’s health-related QOL model to test the structural model of the QOL in elderly with CMP according to sex (Figure 1). The Wilson and Cleary model presents a taxonomy of patient outcomes categorized into five underlying health concepts and proposes specific causal links between these health concepts, and these concepts were influenced by characteristics of both individuals and environments [20]. In this study, subjects were selected as those with CMP as diagnosed by doctors and who experienced pain in musculoskeletal areas in the last six months. First, biological function refers to functions at the level of cells, organs, and organ systems. Because the sample was the community-dwelling elderly, the collection of medical records was limited. Therefore, rather than collecting inaccurate data from participant recall, biological functions were excluded from the hypothetical model. The physical, emotional, and cognitive symptoms perceived by the patient were taken as the second concept. In this study, we measured the intensity of pain caused by MSD. For the third concept, the functional state was taken as the physical, psychological, social, and role function. We examined the degree of disease-specific physical function limitation experienced by the subject. The next linked concept was general health perception; this component is most commonly measured with a single question asking people to rate their health on a Likert scale ranging from poor to excellent [20]. In this study, we measured perceived self-reported health status using a 3-item Likert scale. Lastly, the QOL was taken as a concept at a broad level of abstraction. QOL is subjective well-being, implied a pleasant and unpleasant affect, which was self-evaluated based on the satisfaction of various domains of life. Because we applied a revised model with nonmedical factors removed, the QOL was operationalized to The World Health Organization Quality of Life Brief Version (WHOQOL-BREF) because it focused on the health-related QOL, which is affected by chronic pain and related factors.

### 2.2. Measures

#### 2.2.1. Pain

The intensity of pain was measured using the numeric rating scale [25] to indicate the amount of pain experienced in the last week. The total score ranged from 0 (no pain) to 10 (maximum pain experienced by the person), with higher scores indicating more severe pain.

#### 2.2.2. Functional Limitations

Functional limitations related to musculoskeletal disorders were measured using Western Ontario and McMaster Universities Osteoarthritis Index Korean version (K-WOMAC) [26], which is the translated and modified version of WOMAC developed by Bellamy et al. [27]. K-WOMAC consists of 24 questions in 3 subareas (pain, stiffness, and physical function). In this study, 17 items corresponding to physical function area were used. The scale was a 5-point Likert scale ranging from 0 (none) to 4 (extreme). A higher score corresponds to greater experienced difficulty in the performance of daily physical activities. As a result of the confirmatory factor analysis (CFA) in this study, the fit indices generally met the criteria: χ^2^ = 269.62 (degree of freedom (df)) = 103, *p* < 0.01, goodness-of-fit index (GFI) = 0.89, normed fit index (NFI) = 0.93, Tucker-Lewis index (TLI) = 0.94, comparative fit index (CFI) = 0.96, and root-mean-square error of approximation (RMSEA) = 0.07. The internal consistent coefficient, Cronbach’s α, was 0.95.

#### 2.2.3. Perceived Health Status

The Modified Health Self-Rating Scale, which was developed by McDowell [28], and translated by Kim [29] into Korean, was used in this study. It consists of 3 self-rated components for assessing overall health: Current health status, health status compared to one year ago, and health status compared to the same age. Each item is rated on a 5-point Likert scale ranging from 1 (not very healthy) to 5 (very healthy). The higher the score, the better the self-perceived health status. As a result of the CFA, the fit indices were acceptable (χ^2^ = 64.05 (df = 8, *p* < 0.01), GFI = 0.90, NFI = 0.92, TLI = 0.94, CFI = 0.95, and RMSEA = 0.05), and Cronbach’s alpha was 0.79.

#### 2.2.4. Pain Coping

The pain response was measured using the Pain Response Inventory (PRI) developed by Walker et al. [30] and translated and validated by Yu [31]. The PRI consists of 60 questions that assess the coping response to pain, which can be classified into three types: Active coping, passive coping, and accommodative coping [30]. Each item is measured using a 5-point Likert scale, from 1 (never) to 5 (always). The higher the average score for an item, the more it is used. As a result of the CFA of the measurement in this study, 9 items corresponding to the passive coping type were deleted (χ^2^ = 75.83 (df = 27, *p* < 0.01), GFI = 0.90, NFI = 0.93, TLI = 0.92, CFI = 0.96, and RMSEA = 0.07). Cronbach’s alpha was 0.85 for active coping and 0.86 for accommodative coping.

#### 2.2.5. Social Support

Social support was measured using the Korean version of Multidimensional Scale of Perceived Social Support (MSPSS) [32] developed by Zimet et al. [33]. Social support consists of 12 items, which were categorized into three factors: Family, friends, and medical staff. Each item was measured using a Likert 5-point scale ranging from 1 (very strongly disagree) to 5 (very strongly agree). The total score ranged from 12 to 60 points, with a higher score indicating a higher degree of social support from friends and medical staff. In this study, the CFA of the measurement showed a good fit (χ^2^ = 94.08 (df = 44, *p* < 0.01), GFI = 0.96, NFI = 0.96, TLI = 0.97, CFI = 0.98, and RMSEA = 0.06), and Cronbach’s α was 0.88.

#### 2.2.6. Health-Related Quality of Life

QOL was measured using Korean WHOQOL-BREF [34,35]. This measurement consists of 26 questions: Two items for overall QOL and general health, 7 items for physical health, 6 psychological items, 3 items for social relationships, and 8 items for environment. In this study, 24 questions were used for the structural model analysis, except for 2 items from the overall QOL. Each item was evaluated using a 5-point Likert scale from 1 (not at all) to 5 (completely). The CFA showed an acceptable fit (χ^2^ = 402.66 (df = 177, *p* < 0.01), GFI = 0.89, NFI = 0.87, TLI = 0.90, CFI = 0.92, and RMSEA = 0.07), and Cronbach’s α was 0.92 in this study.

### 2.3. Participants and Study Process

This was a cross-sectional study conducted with the aim of examining the relationship between health-related QOL and related factors in elderly patients with CMP. We applied the Strengthening the Reporting of Observational Studies in Epidemiology (STROBE) statement [36]. Our study is based on data collected by Jeong and Lee [23]. The participants were elderly people aged 65 years or older recruited conveniently from 7 senior welfare service centers located in 2 cities in South Korea. The senior welfare service center provides consultations for the elderly for free or a low rate in addition to facilities necessary for the promotion of health, culture, entertainment, and the welfare of the elderly. The inclusion criteria of the participants were diagnosed with musculoskeletal diseases by a physician, having CMP lasting more than 6 months, and answering “yes” to the two questions “Have you ever been diagnosed with musculoskeletal disease by doctor?” and “Have you experienced CMP in the last six months?” Those who could not complete the questionnaire or who had cognition problems were excluded. The minimum number of samples required for multiple regression analysis was calculated based on the 15 independent variables (effect size = 0.10, α = 0.05, and power = 0.90). A questionnaire was distributed to 360 people considering a 30% dropout rate. The response rate of the questionnaires was 100%, but a total of 307 questionnaires were used for analysis as 53 questionnaires were excluded on the basis of unfaithful responses or missing values. Since the minimum number of samples required to apply the maximum likelihood method for SEM was 300, the sample size in this study was considered suitable for analysis [37].

### 2.4. Ethical Approval

We used data from Jeong and Lee [23], whose study had previously been approved by the Institutional Bioethics Review Board (*** IRB/2019_43_HR) in July 2017. This study was approved by the Institutional Bioethics Review Board in April 2019 for secondary analysis of the earlier data (*** IRB/2017_57_HR). Before commencement of the research, participants were informed about the study objectives and procedures to which they provided written consent. Extracted data were anonymized using identification numbers instead of identifiable information.

### 2.5. Statistical Analysis

Descriptive statistics, including skewness and kurtosis of observational variables, were calculated. Independent t-tests were used to investigate the differences in the study variables between the sexes. To evaluate the validity of the measurement model, factor loading was analyzed using CFA with the maximum likelihood method assuming multivariate normality to estimate the structural model. As a result of evaluating multicollinearity using the tolerance and variance inflation factor (VIF), the tolerance values were all over 0.1, and VIF was less than 10, indicating no multicollinearity between the study variables. For multigroup SEM, the configural invariance of the base model was examined to determine whether it is a suitable model for both elderly women and men with CMP. Afterwards, the measurement weights model was tested for both men and women. Finally, the structural weights model was tested to confirm whether the relationship between each of the potential variables could be equally applied to both groups. We used 200 iterations of bootstrapping to analyze the statistical significance of direct, indirect, and total effects of variables on QOL according to sex. The overall fit of the hypothesized model was assessed using CFI, GFI, NFI, TLI, and RMSEA. To determine the extent to which the estimates were statistically significantly in samples, we calculated 95% confidence intervals. Statistical significance was defined as a two-tailed p-value of <0.05 for all calculations. Statistical analyses were conducted using SPSS WIN 25.0 (Corp, Armonk, NY, USA) and AMOS 25.0 (Corp, Armonk, NY, USA).

## 3. Results

### 3.1. Descriptive Statistics

For multi-group analysis, the normal distribution of pain, K-WOMAC, perceived health status, PRI, MSPSS, and WHOQOL-BREF were tested. All variables were normally distributed, the absolute value of skewness did not exceed 3, and the kurtosis absolute value did not exceed 10. The results of the *t*-test to determine the difference between the variables according to sex are shown in Table 1. Pain (*t* = −3.89, *p* < 0.001) and functional limitation (*t* = −3.65, *p* < 0.001) were more severe in women than in men, and the women perceived their health to be worse than the men (*t* = 2.03, *p* = 0.04). Both active pain coping (*t* = −2.10, *p* = 0.04) and accommodative pain coping (*t* = −3.19, *p* = 0.001) were found to be more commonly used by women. Women received more support from their peers than men (*t* = −3.30, *p* = 0.001).

### 3.2. Validity and Reliability of Measurement Model

Table 2 presents the CFA of the measurement model. CFA was used to evaluate whether multiple indicators in each latent structure can reduce measurement errors and accurately fit the data in the given measurement model. According to the results, the model-fit indices of the hypothesized model did not meet the criteria, with χ^2^ = 272.09 (df = 53, *p* < 0.01), GFI = 0.89, NFI = 0.80, TLI = 0.76, CFI = 0.83, and RMSEA = 0.12. The measurement model was modified to meet the proposed fit indices by deleting items with poor factor loading (less than 0.2). As a result, when the latent construct in terms of the passive response of PRI was deleted, the fit indices of the final modified measurement model met the criteria χ^2^ = 19.62 (df = 42, *p* < 0.01), GFI = 0.94, NFI = 0.90, TLI = 0.90, CFI = 0.93, and RMSEA = 0.08.

The composite reliabilities ranged from 0.63 to 0.89, which were all suitable as they were greater than 0.60, as recommend by Fornell and Larcker [38]. The values for the average variance extracted (AVE) were all greater than 0.25, ranging from 0.37 to 0.75 [37].

### 3.3. SEM and Hypothesis Testing

Table 3 presents the SEM results of the final model of QOL among older people with CMP. We found that 10 of the 13 paths were significant, and all the fit indices (χ^2^ = 96.15 (df = 41, *p* < 0.01), GFI = 0.95, NFI = 0.92, TLI = 0.92, CFI = 0.95, and RMSEA = 0.07) were within the acceptable range. The path from pain coping to pain (β = 0.19, *p* = 0.04) was significant. The paths from pain to functional limitation (β = 0.47, *p* < 0.001) and from pain coping to functional limitation (β = 0.28, *p* < 0.001) were significant, but the paths from social support to functional limitation and from social support to perceived health status were not (β = −0.58, *p* < 0.001). From social support to perceived health status (β = 0.23, *p* = 0.002) was significant, but the path from pain coping to perceived health status was not. A significant and positive relationship was observed between QOL and perceived health status (β = 0.24, *p* < 0.001), pain coping (β = 0.31, *p* < 0.001), social support (β = 0.44, *p* < 0.001), pain (β = −0.15, *p* = 0.01), and functional limitation (β = −0.20, *p* = 0.01).

### 3.4. Multigroup Analysis

Multiple group analysis was based on a modified SEM to examine the statistical differences in factors affecting the quality of life between. In this study, the significance of differences between groups was tested in a hierarchical manner using a model with a constraint on the unconstrained model for measurement weight, structural weights, and measurement residuals.

First, for the baseline model in terms of unconstrained model tests, configural invariance was used to determine whether the number of factors was invariant in both groups. The fit indices of unconstrained model were χ^2^ = 113.509 (df = 82, *p* < 0.001), TLI = 0.95, CFI = 0.97, and RMSEA = 0.03, which means that a similar factor structure was present in both groups. The measurement weights model constrained the factor loadings equally across the groups. The results did not demonstrate a significant difference in chi-square (Δχ^2^ (6) = 10.003, *p* = 0.120), which indicated that the measurement variables affected both men and women similarly. Finally, there was a significant increase in chi-square between the measurement weights model and the structural weights model (Δχ^2^ (13) = 28.837, *p* = 0.007), which means that the relationships between variables across sex groups must be analyzed separately (Table 4).

**Figure 2:**
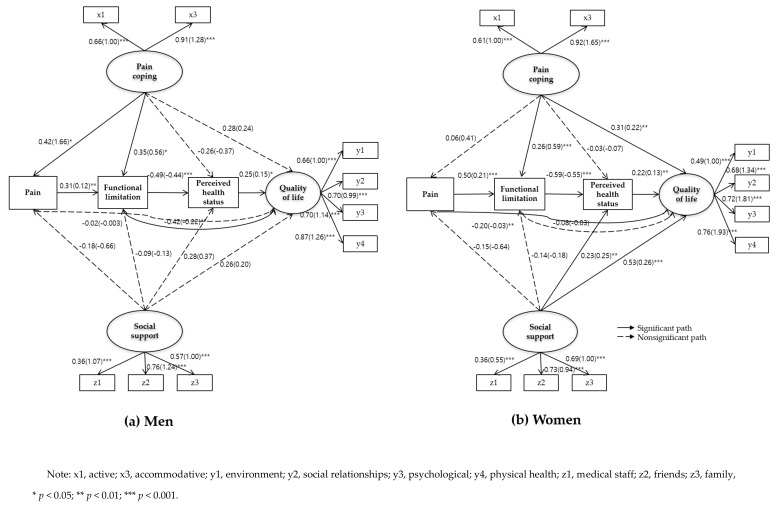
The estimated paths in (**a**) men and (**b**) women.

Figure 2 presents the SEM path coefficients of QOL among elderly men and women with CMP. First, the SEM for elderly men with CMP (Figure 2a) shows a sequential causality from pain to functional limitation (β = 0.31, *p* = 0.002), from functional limitation to perceived health (β = −0.49, *p* < 0.001), and from perceived health status to QOL (β = 0.25, *p* = 0.04). Regarding the relationship between pain coping and social support and other variables, pain coping was not directly related to QOL, but it was associated with pain (β = 0.42, *p* = 0.01) and functional limitation (β = 0.35, *p* = 0.03). However, social support was not related to any variable.

Social support was not related to any variable. Similar to the group of men, the SEM for elderly women with CMP (Figure 2b) showed a sequential causality from pain, functional limitation (β = 0.50, *p* < 0.001), perceived health status (β = −0.59, *p* < 0.001), and QOL (β = 0.22, *p* = 0.009). Pain coping was found to directly affect the QOL (β = 0.31, C.R. = 3.26, *p* = 0.001) and mediate functional limitation (β = 0.26, *p* < 0.001) to affect QOL. Social support also exhibited a direct impact on QOL (β = 0.53, *p* < 0.001), and affected the QOL by mediating perceived health status (β = 0.23, *p* = 0.006).

Table 5 presents the direct, indirect, and total effects of the variables on the QOL under the condition of measurement invariance among elderly men and women with CMP. In men, functional limitations were statistically significant for the direct effect (β = −0.417, *p* = 0.04) and total effect (β = −0.182, *p* = 0.02) on QOL. Pain coping indirectly affected QOL (β = −0.331, *p* = 0.02), but its total effect on QOL was not significant. In elderly women with CMP, the QOL was directly affected by social support (β = 0.528, *p* = 0.02), pain coping (β = 0.306, *p* = 0.04), and perceived health status (β = 0.224, *p* = 0.02). Pain (β = −0.107, *p* = 0.05) and functional limitation (β = −0.132, *p* = 0.02) indirectly affected QOL. Overall, functional limitation (β = −0.539, *p* = 0.02) was a significant factor regarding the total effect on QOL in men. By contrast, social support (β = 0.656, *p* = 0.01) had the greatest total effect on QOL in women, followed by pain (β = −0.310, *p* = 0.02) and perceived health status (β = 0.224, *p* = 0.02).

Lastly, Table 6 presents pairwise comparisons of the path coefficients using critical ratios to identify group differences. We found significant differences between men and women in the three paths: From pain coping to pain (*t* = −1.97, *p* < 0.05), from pain to functional limitation (*t* = 2.02, *p* < 0.05), and from functional limitation to QOL (*t* = 2.52, *p* < 0.05).

## 4. Discussion

In this study, we aimed to construct an SEM based on Wilson and Cleary’s QOL model to analyze the influence of various factors on QOL after establishing measurement invariance across sexes in a sample of Korean elderly aged 65 years or over and experiencing CMP. The results of the SEM were partially consistent with Wilson and Cleary’s model. First, a causal relationship was found between adjacent variables in terms of pain, functional limitations, perceived health status, and QOL in both sexes. Wilson and Cleary’s model empirically examined the causal relationship between adjacent concepts through various subjects with chronic disease [39]. However, the model had not been previously tested in older patients with CMP; in this study, we tested the model in the real world with community-dwelling elderly experiencing CMP and demonstrated its applicability.

Wilson and Cleary’s model empirically examined the causal relationship between adjacent concepts through various subjects with chronic disease [39]. However, the model had not been previously tested in older patients with CMP; in this study, we tested the model in the real world with community-dwelling elderly experiencing CMP and demonstrated its applicability. The results of the relationships in this study were found to be inconsistent with the Wilson and Cleary model. First, we added pathways to Wilson and Cleary’s model, where pain and functional limitations directly affect QOL. Second, pain coping and social support were found to have a direct effect on QOL, but the effects on pain, functional limitation, and perceived health status were inconsistent. In multigroup analysis, we observed differences in the paths between the sexes.

Pain did not directly affect QOL in structural models for men and though a direct influence pathway was established for women; however, we found no statistically significant differences between sexes. The path coefficient from pain to functional limitation, however, was larger in women than men and this difference was considered statistically significant. In this study, pain was more severe in women than in men, which is consistent with the studies of European elderly with CMP [40]. This is related to differences in the perception of pain between the sexes [41]. Therefore, in considering the indirect effects of pain on QOL through functional limitation, pain management needs to be improved in elderly women with CMP.

Functional limitation directly affected QOL more in men than in women, with a statistically significant difference between sexes. Limitations with respect to the activities of daily life considerably impacted the QOL of people with musculoskeletal disorders [42]. In elderly people with hallux valgus, the greater the degree of deformity, the greater the functional limitation, which ultimately affects the quality of life [43]. As these limitations also impact family and social relationships [42], the aim of long-term management to improve the quality of life for patients with musculoskeletal disease should focus on rehabilitation strategies that minimize these functional limitations. In particular, men in Korea have actively played social roles through professional activities for a long time compared with women, which can result in a greater effect on the quality of life through increased negative attitudes due to physical limitations. Therefore, further research is needed to confirm how men and women perceive social and physical functions, and how this is related to quality of life. We found that older women were more restricted in function than older men, which is consistent with the results of a study of CMP in older European adults [40]. Given the better evaluation of physical health by men than women [44], we suggest that an approach that takes into account the differences in the basic functional levels of elderly men and women would be effective.

Pain coping affected pain and functional limitation in men, but in women, pain coping directly affected QOL. However, only the path from pain coping to pain was significantly different between the sexes. Pain coping, an individual characteristic, was found to directly affect the QOL and to affect QOL through pain and functional limitation. The greater the pain coping in patients with spinal stenosis, the higher their subjective walking ability and social function [45]. The decrease in coping strategies in patients with irritable bowel disease (IBD) was a predictor of decreasing QOL [46]. Pain coping is an emotional response that can change through mediation. According to the pain coping styles of patients suffering from chronic low back pain, different beliefs about mood disorders and pain control are reported, suggesting the need for pain coping programs in terms of personal behavior and cognition [47]. Intervention to reinforce strategies for coping with pain in patients with chronic pain resulted in a reduction in the intensity and interval of pain, and a positive change in psychological function [48]. Pain response training programs, including exercise interventions, were conducted in adults over 50 years of age living in the community, resulting in reduced functional disability and pain [49]. Therefore, the implementation of pain response skills training for the elderly with CMP is a major intervention that leads to pain and function improvement, ultimately improving QOL.

According to a previous study, women show passive and maladaptive coping types, whereas men use more adaptive coping patterns [15]. The more active the pain coping strategy, the higher the QOL; patients using accommodative coping exhibit less depression and disability [50]. Conversely, passive pain coping can lead to a negative perception of pain and cause symptoms, such as depression and anxiety, and depression in elderly patients with chronic pain results in decreased QOL [51]. However, in this study, passive pain coping as a sub-factor of PRI was eliminated in the measurement model due to a low path coefficient and the poor fit of the model. The subjects in this study were able to function independently. For instance, their visits to a welfare center implies that their social activities are active to an extent. Korean seniors use the least amount of avoidance pain treatment [52]; hence, we suggest that the avoidance coping strategy in this study was not significant in the construction of the PRI. Carefully testing the cultural validity of the PRI in the Korean population is necessary. The cultural validity of the tools needs to be closely examined. Therefore, to improve the QOL of elderly people with CMP, reinforcing their active or accommodative coping strategies is necessary. Also, further research is needed to identify which types of pain management strategies are more effective according to sex.

Overall, the SEM showed that social support affected QOL but differed structurally between sexes. For women, it was found that social support affects QOL directly as well as indirectly through perceived health status, though the effect was not statistically significant. The examination of gender differences in the QOL of elderly people living alone showed that women visit senior citizens’ welfare centers and actively engage in religious activities more than older men [44]. This means that older women receiving more peer support through these activities compared to older men. In contrast to women, older men resort to their own experiences rather than receive social support. This may enhance their psychological well-being and productivity and reduce depression [53]. In this sense, exploring the differences between men and women in terms of whether the experience of receiving or providing social support affects QOL is necessary.

This study is the first to provide a rationale for customized intervention strategies to improve QOL among elderly Koreans with CMP. However, this study has some limitations that affect the interpretation of the results. Because twice as many women than men were included, the social support in the overall model may reflect female characteristics. Further research is needed using a different sample size and examining sociodemographic factors that can affect social support. The subjects of this study were collected by convenience sampling, so bias may exist in the relationship between study variables due to the inability to control sociodemographic variables. Therefore, in future research, the bias between groups should be controlled using methods, such as the propensity matching score. In this study, variables in terms of biological factors were not included; therefore, we could not sufficiently validate the theoretical model as a whole. We recommend that future studies measure more specific variables associated with musculoskeletal disorders, such as biomarkers or radiological images. Future studies should test the model using different measurement variables reflecting the environmental and personal factors described in the Wilson and Cleary model also. In this study, the passive coping construct was removed from the original version of the pain coping measurement. Thus, further validation is required to establish the external validity and reliability of the Korean version measurement of PRI. Although the causal relationship between concepts was verified in the model, this study is limited by its cross-sectional nature, so in-depth longitudinal studies are needed. Lastly, since cultural differences can affect environmental factors, demographic characteristics, and personal preferences and values, further studies should be conducted with data collected from different countries.

## 5. Conclusions

In this study, we developed a model to describe the QOL of elderly patients with CMP and identified differences in the underlying pathways according to sex. The results show that pain, functional limitation, perceived health status, pain coping, and social support are directly or indirectly related with the QOL. In particular, the relationship among pain, pain coping, functional limitation, and QOL differ between men and women. Based on the results, QOL could be improved using interventions that focus on improving pain coping skills and functional ability would be effective in men. In women, however, interventions that decrease pain should be prioritized, and social support status would be expected to have a considerable impact.

## Figures and Tables

**Table 1:** Descriptive statistics and sex differences of the major variables (*N* = 307).

Variables		Total Sample				Men (n = 101)				Women (n = 206)				*t*
		M	SD	S	K	M	SD	S	K	M	SD	S	K	
Pain		6.24	2.11	−0.08	−0.43	5.59	2.00	0.18	−0.02	6.55	2.10	−0.23	−0.40	−3.885 ***
Functional limitation		30.62	15.18	0.12	−0.67	26.20	13.64	0.29	−0.03	32.79	15.45	−0.02	−0.81	−3.645 ***
Perceived health status		6.63	2.50	0.15	−0.77	7.04	2.18	−0.12	−0.51	6.43	2.62	0.29	−0.79	2.027 *
Pain coping	Active	3.12	0.73	−0.14	−0.24	3.00	0.71	0.01	0.23	3.18	0.74	−0.06	0.14	−2.101 *
	Accommodative	3.00	0.71	−0.28	0.12	2.81	0.76	−0.21	0.26	3.10	0.67	−0.33	0.34	−3.192 **
	Passive	2.32	0.71	0.37	−0.50	2.27	0.72	0.41	−0.39	2.35	0.72	0.48	−0.51	−1.002
Social Support	Medical staff	9.72	4.32	0.37	−0.84	9.45	3.82	0.27	−0.90	9.85	4.55	0.37	−0.91	−0.769
	Friends	12.07	3.80	−0.14	−0.57	11.06	3.52	−0.07	−0.20	12.56	3.84	−0.23	−0.64	−3.301 **
	Family	13.30	4.13	−0.45	−0.40	13.22	3.79	−0.33	−0.32	13.34	4.30	−0.50	−0.44	−0.243
Quality of Life	Physical health	19.83	4.54	−0.09	0.29	20.34	3.70	0.47	3.04	19.58	4.89	−0.14	0.38	1.378
	Psychological	18.75	3.84	0.02	0.54	18.73	3.37	0.60	2.31	18.76	4.06	−0.15	0.06	−0.053
	Social relationships	8.64	1.91	0.04	0.44	8.35	1.77	0.02	1.64	8.78	1.96	0.01	0.07	−1.864
	Environment	25.08	4.69	−0.08	0.91	24.46	4.40	−0.37	1.64	25.40	4.81	−0.01	0.62	−1.728

Note: M, mean; SD, standard deviation; S, skewness; K, kurtosis. * *p* *<* 0.05, ** *p* *<* 0.01, *** *p* *<* 0.001.

**Table 2:** Confirmatory factor analysis of the measurement model.

Latent Variables	Observed Variables	Unstandardized Estimates (95% CI; Lower, Upper)	SE	C.R.	Composite Reliability	AVE
Pain coping	Active	1.442 (1.207, 1.742)	0.185	7.81	0.86	0.75
	Accommodative	-	-	-		
Social Support	Family	1.333 (0.878, 2.545)	0.263	5.08	0.63	0.37
	Friends	1.723 (1.189, 3.193)	0.328	5.26		
	Medical staff	-	-	-		
Quality of life	Physical health	1.292 (1.013, 1.655)	0.123	10.53	0.89	0.67
	Psychological	1.322 (0.997, 1.635)	0.129	10.25		
	Social relationships	0.993 (0.666, 0.963)	0.105	9.51		
	Environment	-	-	-		

Note: CI, confidence interval; SE, standard error; C.R., critical ratio; AVE, average variance extracted.

**Table 3:** The structural equation model of quality of life (QOL) among elderly people experiencing chronic musculoskeletal pain (CMP).

Endogenous Variables	Exogenous Variables	Unstandardized Estimates (95% CI; Lower, Upper)		SE	C.R.	*p*	SMC
**Pain**	Pain coping	0.87 (−0.06, 2.24)		0.37	2.37	0.02 *	0.03
	Social support	−0.40 (−1.40, 0.30)		0.28	−1.44	0.15	
Functional limitation	Pain coping	0.54 (0.11, 0.82)		0.14	3.91	<0.001 ***	0.32
	Pain	0.20 (0.15, 0.25)		0.02	9.51	<0.001 ***	
	Social support	−0.16 (−0.38, 0.08)		0.10	−1.52	0.13	
Perceived health status	Functional limitation	−0.54 (−0.64, −0.45)		0.05	−11.72	<0.001 ***	0.40
	Social support	0.29 (0.08, 0.61)		0.09	3.11	0.002 **	
	Pain coping	−0.14 (−0.46, 0.14)		0.12	−1.15	0.25	
Quality of life	Pain coping	0.23 (0.04, 0.42)		0.07	3.59	<0.001 ***	0.62
	Perceived health status	0.10 (0.05, 0.18)		0.03	3.34	<0.001 ***	
	Social support	0.23 (0.07, 0.38)		0.05	4.30	<0.001 ***	
	Pain	−0.03 (−0.06, 0.007)		0.01	−2.52	0.01 *	
	Functional limitation	−0.08 (−0.15, −0.002)		0.03	−2.59	0.01 *	
	$\chi^{2}$	df	TLI	CFI	RMSEA	NFI	GFI
model fit	96.15	41	0.92	0.95	0.07	0.92	0.95

Note: df, degree of freedom; SMC, squared multiple correlations TLI, Tucker–Lewis index; CFI, comparative fit index; RMSEA, Root Mean Square Error of Approximation; NFI, Normed Fit Index; GFI, Goodness of fit index; *: *p* < 0.05; **: *p* <0.01; ***: *p* < 0.001

**Table 4:** Testing for measurement invariance between sexes.

Model	$\chi^{2}$	df	TLI	CFI	RMSEA	$\Delta \chi^{2}$ (Δdf)	*p*
Unconstrained	113.509	82	0.955	0.972	0.035	-	-
Measurement weights	123.612	88	0.953	0.969	0.036	10.103 (6)	0.120
Structural weights	152.449	101	0.941	0.955	0.041	28.837 (13)	0.007

**Table 5:** Standardized direct, indirect, and total effects of variables on quality of life.

Path	Men			Women		
	Direct Effects	Indirect Effects	Total Effects	Direct Effects	Indirect Effects	Total Effects
Pain coping → Pain	0.418 *	-	0.418 *	0.059	-	0.059
Social Support → Pain	−0.179	-	−0.179	−0.152	-	−0.152
Pain → Functional limitation	0.308	-	0.308	0.504 **	-	0.504 **
Pain coping → Functional limitation	0.353	0.129	0.481 *	0.259 *	0.030	0.289 *
Social Support → Functional limitation	−0.141	−0.055	−0.141	−0.141	−0.077	−0.217 **
Pain → Perceived health status	-	−0.149	−0.149	-	−0.297 **	−0.297 **
Functional limitation → Perceived health status	−0.485	-	−0.485 *	−0.588 *	-	−0.588 *
Social Support → Perceived health status	0.278	0.068	0.346	0.227 **	0.128 **	0.355 **
Pain coping → Perceived health status	−0.260	−0.234 *	−0.493 **	−0.028	−0.170 *	−0.198
Pain → Quality of life	−0.016	−0.166	−0.182	−0.204	−0.107 *	−0.310 *
Functional limitation → Quality of life	−0.417 *	−0.121	−0.539 *	−0.08	−0.132 *	
Perceived health status → Quality of life	0.250	-	0.250	0.224 *	-	0.224 *
Pain coping → Quality of life	0.284	−0.331 *	−0.046	0.306 *	−0.080	0.227
Social Support → Quality of life	0.260	0.148	0.409	0.528 *	0.128 **	0.656 *

Note: *: *p* < 0.05 **: *p* < 0.01.

**Table 6:** Comparison of path coefficients between sexes.

Endogenous Variables	Exogenous Variables	Men					Women					*t*
		Standardized Estimates	SE	C.R.	*p*	SMC	Standardized Estimates	SE	C.R.	*p*	SMC	
Pain	Pain coping	0.42	0.66	2.53	0.01 *	0.11	0.06	0.45	0.68	0.50	0.02	−1.97 *
	Social support	−0.18	0.64	−1.03	0.30		−0.15	0.28	−1.55	0.12		0.34
Functional limitation	Pain coping	0.35	0.26	2.16	0.03 *	0.25	0.26	0.17	3.31	<0.001 ***	0.33	0.04
	Pain	0.31	0.04	3.13	0.002 **		0.50	0.03	8.45	<0.001 ***		2.02 *
	Social support	−0.09	0.24	−0.53	0.60		−0.14	0.10	−1.65	0.10		−0.17
Perceived health status	Functional limitation	−0.49	0.09	−4.96	<0.001 ***	0.35	−0.59	0.06	−10.02	<0.001 ***	0.43	−1.20
	Social support	0.28	0.21	1.74	0.08		0.23	0.10	2.77	0.006 **		−0.45
	Pain coping	−0.26	0.23	−1.65	0.10		−0.03	0.16	−0.38	0.70		1.15
Quality of life	Pain coping	0.28	0.15	1.56	0.12	0.43	0.31	0.08	3.26	0.001 **	0.76	0.05
	Perceived health status	0.25	0.07	2.05	0.04 *		0.22	0.03	2.61	0.009 **		−0.78
	Social support	0.26	0.14	1.43	0.15		0.53	0.06	4.18	<0.001 ***		0.21
	Pain	−0.02	0.02	−0.16	0.88		−0.20	0.01	−2.74	0.006 **		−1.17
	Functional limitation	−0.42	0.07	−3.20	<0.001 ***		−0.08	0.03	−0.92	0.36		2.52 *

Note: * *p* < 0.05, ** *p* < 0.01, *** *p* < 0.001.
